# Stretch Injury of Human Induced Pluripotent Stem Cell Derived Neurons in a 96 Well Format

**DOI:** 10.1038/srep34097

**Published:** 2016-09-27

**Authors:** Sydney A. Sherman, Jack K. Phillips, J. Tighe Costa, Frances S. Cho, Sevan R. Oungoulian, John D. Finan

**Affiliations:** 1 Department of Neurosurgery, NorthShore University HealthSystem, Evanston, IL, United States; 2 Department of Biomedical Engineering, Columbia University, New York, NY, United States; 3 Independent Contractor New York, NY, United States.

## Abstract

Traumatic brain injury (TBI) is a major cause of mortality and morbidity with limited therapeutic options. Traumatic axonal injury (TAI) is an important component of TBI pathology. It is difficult to reproduce TAI in animal models of closed head injury, but *in vitro* stretch injury models reproduce clinical TAI pathology. Existing *in vitro* models employ primary rodent neurons or human cancer cell line cells in low throughput formats. This *in vitro* neuronal stretch injury model employs human induced pluripotent stem cell-derived neurons (hiPSCNs) in a 96 well format. Silicone membranes were attached to 96 well plate tops to create stretchable, culture substrates. A custom-built device was designed and validated to apply repeatable, biofidelic strains and strain rates to these plates. A high content approach was used to measure injury in a hypothesis-free manner. These measurements are shown to provide a sensitive, dose-dependent, multi-modal description of the response to mechanical insult. hiPSCNs transition from healthy to injured phenotype at approximately 35% Lagrangian strain. Continued development of this model may create novel opportunities for drug discovery and exploration of the role of human genotype in TAI pathology.

Traumatic brain injury (TBI) remains a major public health challenge, causing 52,000 deaths and 275,000 hospitalizations annually in the United States^1^. In addition, more than 1 million patients are diagnosed in the U.S. annually with concussion, which promotes neurodegeneration in some individuals^2^. TBI consists of several distinct neurological disorders, including edema, traumatic axonal injury (TAI), contusion and multiple types of hematoma. The study of these diverse disorders as a single disease entity is increasingly seen as a barrier to innovation since each has different pathology^3^. Therefore, this study focuses specifically on TAI. The human brain is soft and heavy. It stretches and shears under its own weight when the head is violently accelerated. This motion hyperextends and injures neurites, causing TAI^4^. TAI is difficult to produce in common lab animals because their brains are lighter than the human brain. More than 30 clinical trials have been conducted in TBI, and all have failed^5^. This may be because pre-clinical tools considered essential in other neurological conditions are unavailable in TBI. Primate modeling in the field stopped decades ago in response to animal welfare controversies and there is no widely accepted, biofidelic, high throughput model. This report presents an *in vitro* model of TAI employing human induced pluripotent stem cell-derived neurons (hiPSCNs) in a 96 well format with the goal of improving the predictive power and efficiency of pre-clinical drug discovery efforts.

hiPSCNs offer unique insight into the pathology of human disease by enabling human *in vitro* models with cells such as cardiomyocytes and neurons that are difficult to ethically obtain by biopsy from human subjects. Batch-to-batch variability in cells produced in academic labs has historically presented significant challenges, but the recent availability of hiPSCNs from commercial vendors with little batch-to-batch variability^6^ has broadened access to this technology and improved reproducibility across labs. iCell neurons from Cellular Dynamics are a mixed population of GABAergic and glutamatergic hiPSCNs expressing multiple ligand gated and voltage gated ion channels^7^. They exhibit spontaneous electrical activity after 7 days in culture^8^,^9^,^10^,^11^ that is comparable to the activity observed with primary rat neuron cultures^12^ or rat organotypic slice cultures^10^ Due to the long life cycle of the human, full functional maturation as evidenced by synchronous, bursting activity requires up to 90 days *in vitro*^13^,^14^. iCell neurons resemble primary rat neurons both in their susceptibility to α-synuclein stress and response to therapy^15^. They also reproduce important human pathologies that cannot be reproduced in rodent cells without genetic modification, such as intercellular tau transmission^16^. hiPSCNs offer unique insight into the genetics of human disease through the creation of patient-specific cells and isogenic cell lines that isolate the influence of a single genetic change. These opportunities are particularly attractive in the study of TBI, which is influenced by genotype in profound and poorly understood ways^17^,^18^,^19^ (reviewed in ref. 20). hiPSCNs have enabled important contributions to the study of neurological disorders^21^,^22^,^23^, but their application to neurotrauma has yet to be demonstrated.

An *in vitro* stretch injury model requires a repeatable, controllable, mechanical insult. Previous models have employed either uniaxial^24^,^25^,^26^ or biaxial^27^,^28^,^29^ loading modes. Biaxial loading induces channel-independent calcium influx via mechanoporation of the cell membrane, which is a widely accepted injury mechanism^30^,^31^,^32^. Uniaxial loading isolates calcium influx due to activation of stretch-sensitive ion channels^33^. Biaxial loading therefore more completely reproduces injury pathology. Also, biaxial stretch loads each cell equally, regardless of orientation, which supports cell-by-cell measures of pathology in high content analysis.

Neurites are highly viscoelastic^34^ so their response to stretch depends strongly on the rate of stretch^28^,^35^. Therefore, realistic strain rates are required to induce realistic phenotypes. Head impact in humans generates a pulse of stress that lasts <20 ms^36^. Air pressure-driven systems can achieve loading ramps in this domain, but engineering challenges associated with the compressibility of air frustrate efforts to expand these systems beyond a 6 well format^37^. Electromagnetic voice coils, which have to date been used primarily to load organotypic slice culture preparations^38^,^39^,^40^, achieve appropriate pulse durations without relying on air pressure and were therefore chosen as the driving element in this model. Biofidelic stretch injury reproduces the clinically observed process of secondary axotomy^24^,^41^,^42^. This process begins with a stretch insult that does not transect the neurite immediately but induces swellings that enlarge and eventually pinch off, transecting the neurite hours or days after the initial insult.

In this study, a custom-built device driven by an electromagnetic voice coil was used to apply a stretch insult to hiPSCNs in a 96 well format. Fluorescent images of intact cells in culture were analyzed to determine cell viability along with several measures of altered neurite morphology. A range of strains were applied to determine the response of these indicators to mechanical insult.

## Results

In quality control testing of the flatness of 10 plates, 7 had no failed wells, 2 had a single failed well and one had 4 failed wells. These results are similar to those observed with commercial plates. In all cases, the failed wells were in the edge rows/columns which were not used for cell culture in experiments (see Methods).

**Figure 1:**
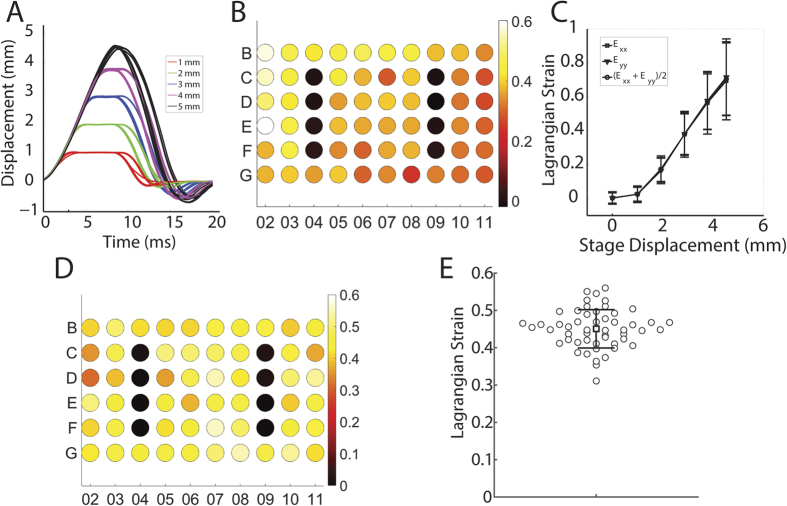
Kinematics of the injury device. (**A**) Stage displacement histories over 10 pulses at a range of target amplitudes. (**B**) Average strain in each well in misaligned configuration with displacement of 2.9 mm (n = 5 measurements per well, average standard error per well = 0.056). Note that C4, D4, E4, F4, C9, D9, E9 and F9 are uninjured control wells. (**C**) The Lagrangian strain in the membrane increased with increasing stage displacement (n = 200–260 wells over 10 plates, bars = standard deviation). (**D**) Average strain in each well in optimally aligned configuration with displacement of 3.3 mm (n = 5 measurements per well, average standard error per well = 0.029). (**E**) Distribution of strains in optimally aligned configuration (circle = average strain in a single well location, square = average strain across all well locations, error bars = 1 standard deviation).

The injury device generated a rapid, highly repeatable, stage displacement history (see Fig. 1A and Table 1). The post array sits in an aluminum block on the injury device that accommodates set screws for optimal alignment with the stage. When these set screws were omitted, a modest misalignment was introduced that created systematic variation in the membrane strain across the plate, with the highest strains on the right side (see Fig. 1B). This configuration was used for dose response experiments and the associated systematic variation was advantageous because it eliminated gaps in the spectrum of strains that would otherwise emerge between different levels of stage displacement (see Fig. 1C). The strain was negligible for the lowest amplitude of displacement tested but rose in an approximately linear manner at higher levels of displacement (see Fig. 1C). The strains in the x and y direction were indistinguishable, indicating that the strain field was equibiaxial^28^. Since low variation across the plate is desirable for applications other than dose response studies, membrane strain was also characterized with optimal alignment. This eliminated systematic variation across the plate (see Fig. 1D). The remaining random variation across well locations created a distribution with a mean of 0.45 and a standard deviation of 0.051 (see Fig. 1E). The average standard deviation of strains at a given well location over multiple tests on different plates was 0.065.

**Figure 2:**
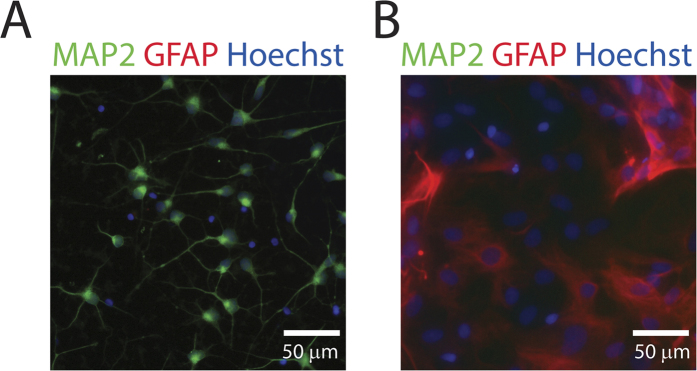
Purity of iCell neurons. (**A**) Representative image of uninjured iCell neurons stained with MAP2, GFAP, and Hoechst 33342 demonstrating the absence of astrocytes. (**B**) Rat astrocyte culture stained and imaged with an identical protocol as a positive control.

There is no evidence of astrocyte contamination in iCell neuron cultures (see Fig. 2). Fixed iCells were immunostained with microtubule-associated protein 2 (MAP2), glial fibrillary acidic protein (GFAP), and the nuclear marker Hoechst 33342. 818 MAP2-positive cells were detected across 6 images but no GFAP-positive cells were detected. Separate cultures of rat astrocytes were stained and imaged simultaneously using an identical protocol to control for errors in GFAP labeling (see Fig. 2B).

**Figure 3:**
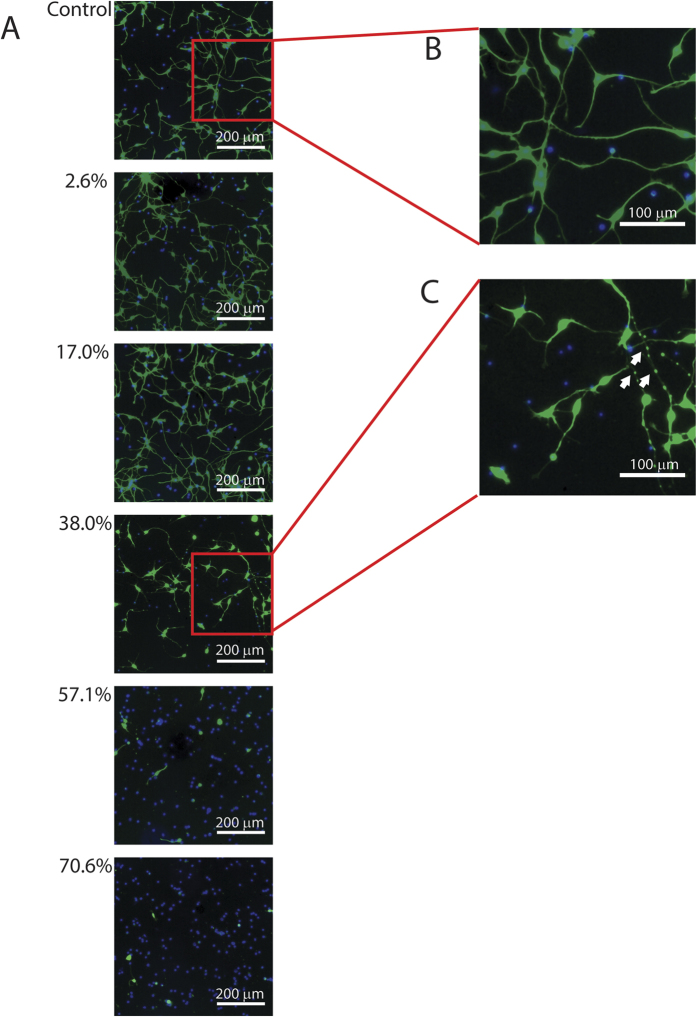
Evolution of injury phenotype with increasing strain. (**A**) Representative images of neurons stained with calcein AM (green) and Hoechst 333342 (blue) 4 hours after injury at various levels of strain. As strain increases, the neurite network becomes less extensive and the number of calcein-AM negative nuclei increases, indicating cell death. (**B**) In the control condition, neurites have a large, constant thickness. (**C**) In injured neurons, neurites are shorter and thinner with beads (see white arrows) distributed along their length.

The injury phenotype increased with increasing strain (see Fig. 3). At the time of injury, the iCell neurons were well-attached and established extensive neurite networks on the silicone-bottomed plates. 4 hours after injury, there was negligible evidence of injury at or below the 17% strain level. A clear injury phenotype emerged at the 38% strain level and rapidly approached saturation at higher strains. The injury phenotype had three components: cell death, shortening of neurites, and changes in neurite shape. Control neurites were thick and uniform (see Fig. 3B) while injured neurites were thin with round beads distributed along their length (see Fig. 3C).

**Figure 4:**
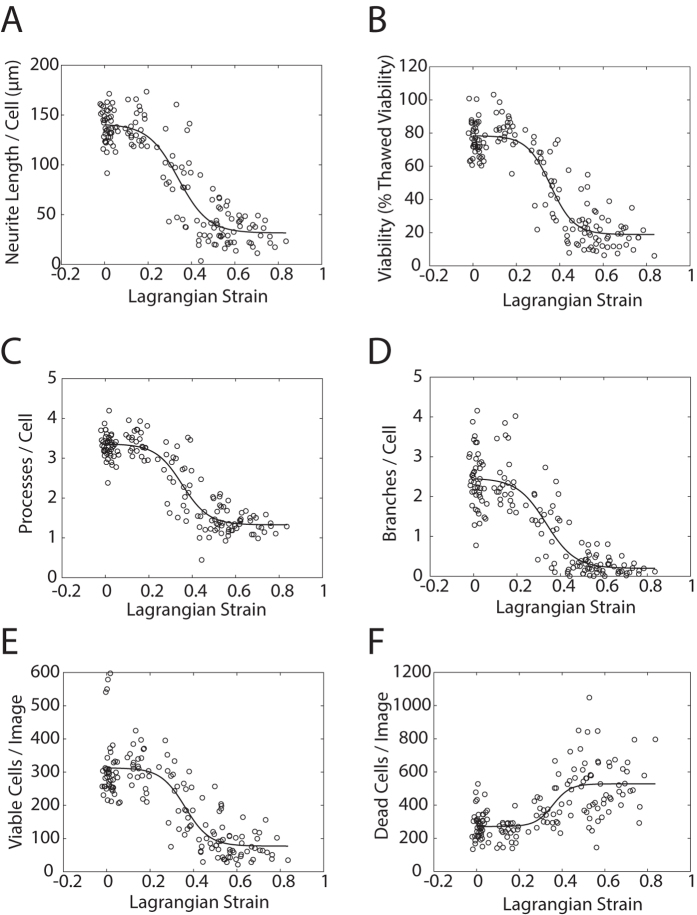
Injury phenotypes increase with increasing strain. (**A**) Mean neurite length per cell declines with increasing strain. (**B**) Cell viability declines with increasing strain. (**C**) Processes/cell declines with increasing strain. (**D**) Branches/cell declines with increasing strain. (**E**) Viable cells/image declines with increasing strain. (**F**) Dead cells/image increases with increasing strain. Injury metrics are plotting against well-specific strain. For phenotype measurements, n = 160 wells over 5 plates. For strain measurements, n = 800 wells over 50 plates (i.e. each point represents the average of 5 measurements (average standard error = 0.056). In both cases, the fit line represents a generalized logistic regression of the data (see Methods). Estimates for the coefficients of each fit along with confidence intervals and R^2^ values are presented in Table 2.

Neurite length and cell viability declined with increasing strain and are distributed in a sigmoidal fashion with respect to strain (see Fig. 4A,B). In the low strain domain (<0.2), there is no obvious increase in injury with increasing strain. In the intermediate strain domain (0.2 < strain < 0.4), injury increases sharply with increasing strain and in the high strain domain (>0.4), the level of injury saturates and becomes strain insensitive again.

Cell viability and several measures of neurite morphology were calculated in each well (see Table 2). A generalized logistic function was fit to these datasets to quantify the variation of phenotype with strain (see Methods). The best fit parameters for this function are presented in Table 2. E_t_, the transition strain, was relatively consistent across the 9 different injury metrics computed, ranging between 0.305 and 0.361.

**Figure 5:**
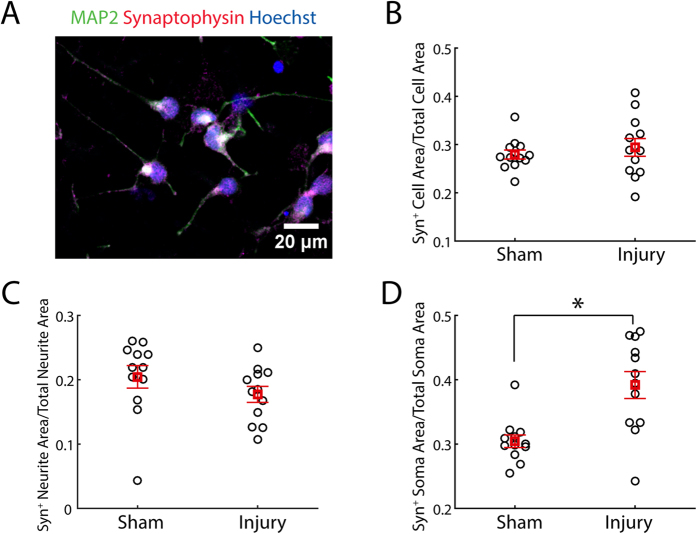
Injury with 2 mm stage displacement alters synaptic density. (**A**) Representative image of uninjured iCell neurons stained with MAP2, Synaptophysin, and Hoechst 33342. Synaptophysin staining was punctate and distributed across soma and along neurites. (**B**) Injury did not change the ratio of synaptophysin positive cell area to total cell area (n = 12). (**C**) Injury did not significantly change the ratio of synaptophysin positive neurite area to total neurite area, although there was a modest downward trend (n = 12). (**D**) Injury significantly increased the ratio of synaptophysin positive soma area to total soma area (n = 12, *=t-test with significance criterion Bonferroni corrected to p < 0.05/3, bars = standard error).

iCell Neurons stained positively for synaptophysin. (see Fig. 5). Injury with 2mm stage displacement did not significantly alter the synaptophysin density in cells overall. However, when the somatic and neuritic compartments were considered in isolation, injury was found to significantly increase somatic synaptophysin density. Injury caused a downward trend in neuritic synaptophysin density, but this trend was not statistically significant.Synaptophysin labeling of synapses revealed the conventional punctate organization (see Fig. 5A). However, it was not appropriate to count synaptophysin-positive puncta per unit length of neurite in this system because the injury induced a beaded morphology in neurites that caused uniform stain distribution to appear punctate. Therefore, synapse distribution was quantified by measuring the ratio of synaptophysin positive cell area to total cell area. Stretch injury did not alter this ratio (Fig. 5B). To further investigate this finding, the cell domain was split into somatic and neuritic domains and the ratio of synaptophysin positive area to total area was computed for each domain. There was a downward trend in the synaptophysin positive area of the neurites, although this trend was not statistically significant (Fig. 5C). By contrast, there was a statistically significant increase in the synaptophysin positive area of the soma (Fig. 5D, t-test with significance criterion Bonferroni corrected to p < 0.05/3).

## Discussion

This model applied a repeatable, mechanical insult to hiPSCNs cultured in a 96 well format and induced a dose dependent, injury phenotype reproducing important aspects of clinical TAI neuropathology. The injury phenotype in some ways resembled that previously reported for *in vitro* stretch injury of rodent neurons and slice cultures. For example, multiple injury metrics transitioned from uninjured to severely injured levels at approximately 30–35% strain (see Table 2). These results agree with previous reports of biaxial *in vitro* models employing rat pup, organotypic, hippocampal, slice cultures, which found that a severe injury phenotype emerged either at 35% strain^40^ or between 20% and 50%^38^. 30% biaxial strain permeabilized embryonic rat neurons *in vitro*^29^. On the other hand, there are potentially significant differences between the response of hiPSCNs to mechanical insult and that previously reported for rodent neurons. For example, a study of embryonic rat neurons reported elevated calcium signaling at both 30% and 50% strain but no change in cell death at either strain level^33^. This study found that cell viability fell when strain reached 36% (see Table 2). This distinction may be attributable to differences in study design, but may also indicate human-specific pathology. This model is the first *in vitro* stretch injury model to employ hiPSCNs. This is significant because it allows patient-specific and isogenic cell lines (tools that have generated unique insights into other neurological disorders)^22^,^23^ to be applied to the study of neurotrauma. Also, it is the first 96 well model applying stretch at rates relevant to neuronal stretch injury. Realistic loading rates are essential because neuronal stretch injury is highly sensitive to loading rate^34^. Finally, it is the first model of stretch injury to quantify neurite morphology as an injury metric (this approach has only previously been used in the study of soluble neurotoxins^43^).

**Figure 6:**
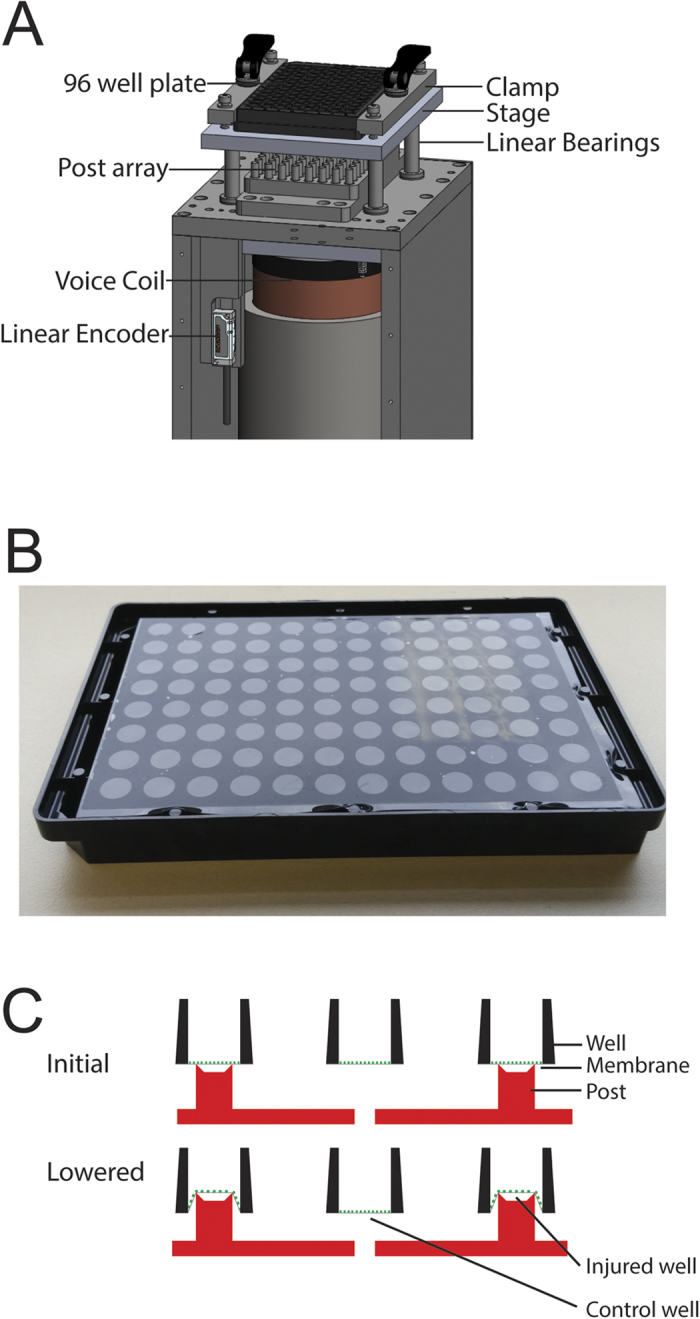
The *in vitro* neuronal stretch injury model. (**A**) The injury device consists of a stage positioned above an array of Teflon-coated, aluminum posts and driven vertically by an electromagnetic voice coil. (**B**) The silicone-bottomed plate consists of a commercially-distributed plate top covalently bonded to a sheet of silicone. Since no sandwich construction or air tight gaskets are employed, the standard geometry of the 96 well plate is preserved. (**C**) Schematic depiction of the injury process. The plate and post array are shown in cross section. Initially, the plate is positioned so that the silicone membrane touches the posts. To induce injury, the plate is lowered, stretching the membrane over the rims of the posts. Posts can be omitted from the post array to create unstretched, control wells.

In this study, the silicone cell culture membrane was indented with a rigid post to induce strain. This approach has important advantages over applying air pressure to induce biaxial strain^44^. Cylindrical indentation of the membrane creates a spatially homogeneous, equibiaxial, strain field. This assertion is supported by theory^45^,^46^ and by the fact that the average Lagrangian strain in the X and Y directions were very similar at all levels of stage displacement (see Fig. 1B). This property of the model means that every cell in culture over the head of the post sees the same mechanical insult, regardless of its position or orientation. In biaxial air-driven systems, the circumferential strain peaks at the center and declines to zero at the edge so the strain environment varies continuously across the well bottom^47^. Also, the conventional geometry of a 96 well plate was retained because inducing deformation without air pressure eliminated the need for gaskets or other special features on the plate bottom (see Fig. 6B). These plates can therefore be manipulated and imaged using existing machinery for high throughput experiments. The relationship between stage displacement and strain in the silicone membrane was linear across all the displacement values tested except for the lowest value, which exhibited almost no strain. This may indicate imperfect zeroing at the start of the displacement pulse or it may indicate a stiction interaction between the silicone and the posts in which deformation begins only after a finite level of tension has been established.

Morphological biomarkers of injury were employed instead of conventional molecular biomarkers such as amyloid precursor protein. This approach has previously been used to study neurotoxicity^48^,^49^ but this is its first application in a neuronal stretch injury model. This high content approach renders the experiment independent of any particular hypothesis about the molecular mechanism of injury and is therefore preferred in drug discovery studies^50^. Morphological metrics integrate the effects of multiple biochemical processes into a single outcome, which is particularly useful in a multi-modal disorder such as TAI. Degeneration of neurites *in vivo* disrupts neural networks and is therefore directly relevant to neurological deficits. The R^2^ value for the fit of neurite length/cell against strain was higher than that for cell viability, suggesting that the former is the more sensitive injury metric (see Table 2).

Mechanical injury modestly perturbed synapse distribution. The reason for an increase in somatic synaptic density with injury is unclear but it is worth noting that injury may exert a selective effect i.e. cells with higher somatic synaptic density may be more likely to survive mechanical insult.

This study is subject to several important limitations. As an *in vitro* model, it omits key features of the *in situ* condition, including the presence of other cell types and the three dimensional environment. Therefore, this model functions as a first step towards increasingly biofidelic human *in vitro* models incorporating these and other features of the *in situ* condition. Brain organoid technology is advancing rapidly^51^, and *in vitro* mechanical injury of neurons in three dimensional culture has been demonstrated^52^ (although extension of quantitative, automated, high content imaging to three dimensions is non-trivial). The model does not currently include any of the numerous other stressors that accompany mechanical stress in clinical TBI, such as oxidative stress, inflammation, and excitotoxicity. However, this can also be viewed as an advantage since it allows the experimenter to control these processes directly and reconstruct them using soluble agents^53^. Also, hiPSCNs are immature at the short culture time points employed in this study, with a gene expression profile typical of a neonate^7^. However, functional maturation can be achieved by prolonged (~90 days) culture^13^ and co-culture with astrocytes^14^. Prior neuronal stretch injury models have employed embryonic rodent neurons^25^,^29^,^33^, murine cell lines^26^,^27^ or human cancer cell lines^24^,^54^, so the feasibility of *in vitro* stretch injury to adult neurons from any source has yet to be demonstrated. Also, it is worth noting that approximately 1,850 children <1 year of age are admitted to U.S. hospitals with abusive head trauma every year so neonatal neurotrauma is an urgent and understudied clinical challenge^55^. While the 96 well format makes efficient use of expensive hiPSCNs, true high throughput drug screening will require greater consistency and this is the focus of ongoing development.

In summary, this paper presents a novel, human, *in vitro* model of TBI in a 96 well format. The model applies a realistic strain and strain rate to human cells to maximize biofidelity. The biomechanical insult is a spatially homogeneous, equibiaxial strain, and the outcome measures are derived from high content analysis of cell viability and morphology. The model induced a dose response relationship between mechanical insult and injury across several different injury metrics that is both internally consistent and consistent with previous findings in other *in vitro* models.

## Methods

### Device Description

The plate stretching device consists of a stage mounted on linear bearings above an array of Teflon-coated aluminum posts, each of which can be removed to create uninjured wells as controls (see Fig. 6). The stage is driven vertically by a LA43-67-000A voice coil actuator (BEI Kimco) with a 15 mm stroke and a peak theoretical acceleration of 2758 ms^−2^. Vertical displacement of the stage is monitored by a T1031-30A optical encoder with 0.1 μm resolution (Renishaw). The system is controlled and monitored using a rack-mounted computer. This computer hosts a cRIO 9024 Real Time PowerPC Controller that sits in a cRIO 9113 chassis and runs a real-time operating system (National Instruments). The cRIO 9024 runs the proportional - integral - derivative control loop that receives displacement information from the optical encoder and regulates the current supplied to the voice coil by a Xenus XTL servo drive (Copley Controls). The force generated by the voice coil is proportional to the current, closing the feedback loop that controls stage displacement. The control loop was tuned using automated routines supplied by the respective manufacturers. A dedicated LabVIEW code specifies the duration and magnitude of the displacement pulse. A separate code is used to record the displacement history at 5000 Hz via an NI 9411 digital input module mounted in the NI 9113 chassis (National Instruments).

### Biomechanical Characterization

The relationship between the stage displacement and the strain in the well bottom was characterized using high speed video imaging of cell-free plates undergoing stretch. 60 wells, from position B2 to position G11, were characterized in this way (note that rows A and H and columns 1 and 12 were not used in experiments to eliminate possible confounds due to edge effects on cellular responses). A 1.5 mm diameter dot was stamped in permanent marker ink in the center of each well using a custom-fabricated, 3D printed stamp. Plates were positioned in contact with the post array as for cell injury experiments and stretched with 0.98, 1.94, 2.87, 3.82 and 4.67 mm amplitude stage displacements. High speed video of the well bottom was recorded at 1000 frames per second and 1280 × 1024 pixel resolution from 30 wells (6 rows and 5 columns) during each stretch experiment using a Fastcam Mini UX50 high-speed camera (Photron) and a flat dome light (CCS Inc). 10 plates were stretched at each peak stage displacement level and half of the plate was imaged each time so that 5 independent measurements could be made in each well at each level of peak stage displacement. Images of the well bottoms before onset of stretch and at peak stretch were selected for analysis. The height and width of the stamped spot in each well was measured manually using a Matlab script custom written for the purpose to quantify the deformation of the well bottom. Shear deformation was neglected in our analysis (see below) because it has repeatedly been shown to be negligible in previous investigations of this type of membrane strain^45^,^46^,^56^. Also, direct measurements of axial strain in perpendicular directions were indistinguishable (see Fig. 4), supporting the assumption of negligible shear strain.

The Lagrangian strains in the x and y directions, E_xx_ and E_yy_, in the plane of the well bottom were defined using equation (1)^57^.


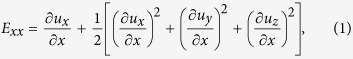



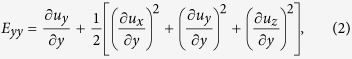


where u is the change in length in the subscripted direction.

Shear deformation was neglected in this analysis, leading to the following simplified expressions:


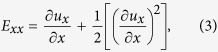



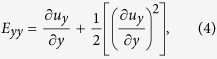


Since neurons are randomly oriented in culture in this model, E_xx_ and E_yy_ are equally important so the Lagrangian strain, E, for a given well was defined as the average of the two.

### Plate Fabrication

Silicone membranes (Specialty Manufacturing Inc.) were covalently bonded to 96 well plate tops (see Fig. 6B) using a protocol adapted from Sukara *et al*.^58^. Briefly, 96 well plate tops were activated for 60 seconds using a plasma cleaner then submerged in 1.5% (3-Aminopropyl) triethoxysilane for 20 minutes. Plates were rinsed twice in deionized water and dried with pressurized air. Silicone membranes were cut into 7.5 × 11 cm rectangles and activated for 60 seconds in plasma cleaner. Plate bottoms and silicone membranes were aligned and clamped together for one hour using custom-built clamps. They were cured for a further 24 hours before use. Complete plates were sterilized in 70% ethanol for 15 minutes then rinsed in sterile water for 5 minutes before cell culture.

### Cell Culture

Neurons derived from human iPSCs were supplied by Cellular Dynamics International under the brand name iCell neurons. Neurons were seeded at 33,750 cells/cm^2^ on to silicone-bottomed plates and maintained in proprietary cell culture media supplied by the vendor. Plates were treated with 0.01% poly-L-ornithine prior to seeding. Neurons were seeded in growth media containing 3.3 μg/ml laminin. For immunofluorescence, neuron density was increased to 67,500 cells/cm^2^ and laminin concentration was increased to 50 μg/ml to minimize losses during the repeated washing required by the immunofluorescence protocol. Plates were incubated at room temperature for 20 minutes after seeding to promote attachment and then maintained at 37 °C, 5% CO_2_.

### Injury

Neurons were injured 48 hours after seeding. The zero position at which the plate touches the post array was established at the start of the experiment using a cell-free plate and a camera positioned above the post array to directly observe contact between the posts and the silicone membrane. Posts were lubricated with corn oil prior to each injury. To create stretch injury, a plate was clamped into the stage and lowered to the zero position. The plate was rapidly displaced down on to the post array and back to its initial position to stretch the membrane. After injury, plates were returned to the incubator. Posts were omitted at positions C4, D4, E4, F4, C9, D9, E9 and F9 to create uninjured control wells.

### Live Cell Image Acquisition

Neurons were incubated for 5 minutes with 1 μg/ml Hoechst 33342 (Sigma, St. Louis, MO) and 2 μg/ml Calcein AM at room temperature 4 hours after injury and imaged immediately. Imaging was performed with a 10x magnification, 0.3 NA Plan Fluor lens on an Eclipse TE2000-U microscope (Nikon). The microscope was maintained at 37 °C during imaging using a Plexiglas enclosure fed by a thermally regulated air supply. All images were acquired near the center of the well in the domain above the rigid cylinder at the time of injury.

### Immunofluorescent Imaging

Cells were fixed for 15 minutes in 4% formaldehyde in D-PBS at room temperature. Cells were washed with D-PBS and received 0.3% Triton-X (Sigma) in D-PBS for 5 minutes at room temperature. Cells were washed with D-PBS and incubated for 1 hour with 5% donkey serum in D-PBS at room temperature. Cells were labeled with 10 μg/ml MAP2 (abcam11267) and 1/1000 diluted GFAP (abcam7260) or 1/1000 Anti-Synaptophysin 1 (Synaptic Systems) primary antibodies in D-PBS with 5% donkey serum overnight at 4 °C. Cells were washed with D-PBS and received 10 μg/ml Alexa Flour 594 (Thermo Fisher) and 10 μg/ml Alexa Flour 488 (Thermo Fisher) secondary antibodies in D-PBS with 5% donkey serum for 1 hour at room temperature. Cells were washed with D-PBS and stained with 3 ng/ml Hoechst 33342 (Sigma) in D-PBS with 5% donkey serum for 10 minutes at room temperature and imaged immediately.

All immunofluorescent imaging employed a 20x, 0.75 NA Plan Apo lens on a Nikon Eclipse Ti microscope. Hoechst MAP2 GFAP labeled images were illuminated with a widefield epifluorescent X-Cite 120 LED lamp and captured using an Andor Zyla scMOS camera to create a 2048 × 2048 pixel image with a 0.33 μm pixel size. Hoechst was excited via a 361–389 nm bandpass filter and emission was collected through a 435–485 nm bandpass filter. Alexa 488 was excited via a 485–505 nm bandpass filter and emission was collected through a 525–555 nm bandpass filter. Alexa 594 was excited via a 540–580 nm bandpass filter and emission was collected through a 605–665 nm bandpass filter. Hoechst MAP2 synaptophysin images were captured using a Nikon C2 confocal microscope to maximize resolution of sub-cellular structure. Hoechst was excited with a 405 nm laser and emission was collected via a 428–463 nm bandpass filter. Alexa 488 was excited with a 488 nm laser and emission was collected via a 500–550 nm bandpass filter. Alexa 594 was excited with a 640 nm laser and emission was collected via a 575–625 nm long pass filter (these unconventional settings were used because they yielded better signal-to-background ratio in practice than conventional red channel settings). Confocal images were 2048 × 2048 pixels with 0.2 μm Nyquist sampled pixel size.

### Image Analysis

**Figure 7:**
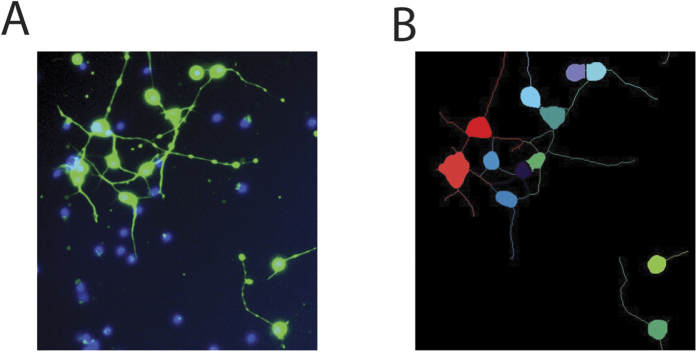
Cell segmentation. (**A**) Neurons injured with 38% strain and imaged 4 hours post injury with calcein AM (green) and Hoechst 33342 staining (blue) (**B**) Automated segmentation of the image into cell bodies and neurites. Note that beads on neurites are rejected as cell bodies based on their size and the absence of Hoechst-positive nuclei. Extracellular nuclei are rejected as cell bodies based on the absence of calcein AM staining.

Live cell images were preprocessed with CellProfiler software^59^. A 3 × 3 median filter was applied to the raw images and uneven, background illumination was corrected. MetaXpress software (Molecular Devices) was used to segment individual cells and compute outcome measures including viable cells/image, dead cells/image, neurite length, process number, and branch number (see Fig. 7).

Immunofluorescent images of cells labeled with synaptophysin, MAP2, and Hoechst were analyzed in Cell Profiler. A 2 × 2 median filter was applied to all channels and the MAP2 channel was thresholded using the Background method^59^ to create a binary image of the area occupied by cells. A morphological erosion operation was applied to eliminate neurites from this image and create a binary image of the soma. The soma image was dilated by 3 pixels and subtracted from the cell image to create a binary image of the neurites. Each of these three domains: cell, soma, and neurites, was applied separately as a mask to the synaptophysin channel. The pixels within the mask were automatically thresholded using the maximum correlation thresholding method with 1 pixel Gaussian smoothing^60^ to identify the synaptophysin positive pixels within each domain. The ratio of synaptophysin positive area to total area in each domain was used to quantify the density of synaptophysin.

### Statistical Methods

A generalized logistic function of the form


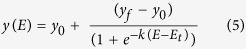


was fit to the injury metric data. In equation (5), y is the injury metric, E is the average of E_xx_ and E_yy_ as defined in the Methods, y_0_ is the zero strain value of the metric, y_f_ is the minimum value which the metric asymptotically approaches at high strain, k is a rate constant governing the transition between the initial and minimum asymptotes, and E_t_ is the transition strain (i.e. the strain value at which the metric is half way between its initial and minimal values). Equation (5) was fit iteratively to the experimental data with the Levenberg – Marquardt algorithm using SPSS v.22 statistics software (IBM) to determine parameter estimates, confidence intervals, and R^2^ values. Initial values of parameters were estimated by visual inspection of scatter plots.

For comparison of synaptophysin density measures, a t-test was used. Since three comparisons were made, the conventional p < 0.05 threshold for significance was Bonferroni corrected to p < 0.05/3.

## Additional Information

**How to cite this article**: Sherman, S. *et al*. Stretch Injury of Human Induced Pluripotent Stem Cell Derived Neurons in a 96 Well Format. *Sci. Rep.* **6**, 34097; doi: 10.1038/srep34097 (2016).

## Figures and Tables

**Table 1:** Repeatability of stage displacement pulse.

Prescribed Amplitude (mm)	True Amplitude (mm)	Duration (ms)
1	0.995 ± 0.0059	11.9 ± 0.56
2	1.93 ± 0.0029	13 ± 0.59
3	2.86 ± 0.013	13.7 ± 0.48
4	3.77 ± 0.021	14.3 ± 0.52
5	4.52 ± 0.049	15.1 ± 0.53

**Table 2:** Estimated coefficients for a generalized logistic regression (see equation 5 for definition of parameters) of the relationship between various injury phenotypes and mean strain.

Phenotype	y_0_	y_f_	k	E_t_	R^2^
Neurite Length/Cell (μm)	141 (134.3, 147.7)	31.55 (23.22, 39.88)	14.43 (8.819, 20.03)	0.339 (0.312, 0.366)	0.834
Cell Viability (% Thawed Viability)	78.44 (75.28, 81.59)	18.91 (14.66, 23.16)	18.43 (10.88, 25.98)	0.357 (0.335, 0.380)	0.828
Processes/Cell	3.36 (3.247, 3.473)	1.327 (1.175, 1.48)	16.43 (9.841, 23.01)	0.353 (0.329, 0.378)	0.828
Total Neurite Length (μm)	43411 (40824, 45998)	2645 (−237.6, 5527)	16.46 (9.47, 23.46)	0.322 (0.296, 0.349)	0.819
Branches/Cell	2.462 (2.281, 2.643)	0.199 (−0.018, 0.417)	15 (7.265, 22.73)	0.334 (0.3, 0.369)	0.745
Total Processes	1063 (985.8, 1141)	103.3 (8.95, 197.7)	17.24 (7.172, 27.32)	0.337 (0.303, 0.37)	0.708
Viable Cells/Image	312.8 (294.3, 331.2)	77.03 (51.43, 102.6)	17.8 (7.213, 28.38)	0.361 (0.328, 0.395)	0.689
Total Branches	801.5 (705, 898.1)	15.94 (−79.97, 111.8)	15.8 (4.113, 27.49)	0.305 (0.255, 0.355)	0.589
Dead Cells/Image	271.1 (239.9, 302.3)	528.2 (489.6, 566.9)	24.58 (−1.24, 50.4)	0.356 (0.313, 0.4)	0.459

Values in parentheses are 95% confidence intervals.
